# Selective embolisation of an idiopathic bronchial artery pseudoaneurysm presenting with recurrent laryngeal nerve palsy: a case report

**DOI:** 10.1186/s42155-024-00474-2

**Published:** 2024-08-14

**Authors:** R. Copping, N. Ng, S. Osman

**Affiliations:** 1 Interventional Radiology Department, Liverpool Hospital, New South Wales, Australia; 2 UNSW Medicine and Health, South West Sydney Clinical Campuses, University of New South Wales, Sydney, Australia

**Keywords:** Bronchial artery pseudoaneurysm, Bronchial artery aneurysm, Embolisation, Bronchial artery embolisation, Recurrent laryngeal nerve palsy

## Abstract

**Background:**

Bronchial artery pseudoaneurysms (BAP) or aneurysms (BAA) are rare, potentially life-threatening and remain poorly understood. They are most commonly idiopathic but may be associated with a number of other disease processes. Bronchial artery embolisation (BAE) is considered the first line treatment while surgical techniques are reserved for patients with a clear contraindication to embolisation or where anatomical factors preclude an endovascular approach.

**Case presentation:**

We present an interesting case of a 56 year-old male presenting with an idiopathic unruptured right BAP causing clinical and radiological signs of left recurrent laryngeal nerve (RLN) palsy. He was otherwise clinically well with no other reported symptoms and no significant past medical history. There were no significant findings on work-up and investigation. He was ultimately treated successfully with selective transarterial coil embolization of the right bronchial artery. This is an atypical presentation of a rare clinical entity and has not previously been published in the literature to our knowledge.

**Conclusions:**

BAPs and BAAs are highly variable in their presentation, ranging from incidental asymptomatic findings to catastrophic haemorrhage, depending on their location and if they are contained or ruptured. Timely diagnosis and referral to facilitate urgent embolisation is essential to prevent potentially serious clinical sequelae. Endovascular treatment in the form of BAE is considered first line.

## Introduction

Bronchial artery pseudoaneurysms (BAP) are rare, potentially life-threatening and remain poorly understood [1]. The terms bronchial artery aneurysm (BAA) and pseudoaneurysm are used interchangeably as the underlying aetiology and pathogenesis are often unknown and not able to be distinguished and they share the same management. They confer high risk of rupture and therefore high mortality. The true incidence is unknown, but it has been reported on less than 1% of selective bronchial angiograms [2]. Presentation is dependent on location and ruptured status, ranging from an incidental finding in an asymptomatic patient to catastrophic haemorrhage resulting in shock.

We present an interesting and atypical presentation of an unruptured right BAP presenting with left recurrent laryngeal nerve (RLN) palsy, subsequently treated successfully with selective bronchial artery embolisation (BAE) with microcoils.

## Case report

A 56 year-old male patient presented with a four-week history of dysphonia (voice hoarseness) and eventual and dysphagia, prompting presentation to his local doctor. He denied any cough, haemoptysis or constitutional symptoms and had no signs of infection. He had no prior history of trauma, surgery, lung disease, vasculopathy or malignancy. There was no personal or family history of connective tissue disease. His only relevant past medical history was idiopathic thrombocytopaenia, with a platelet count as low as 50 × 10^9^/L. On initial investigation, he had a platelet count of 100 × 10^9^/L and positive antinuclear antibodies (ANA) and negative extractable nuclear antigens (ENA). All other blood tests were normal. Repeated blood cultures were negative.

**Fig. 1:**
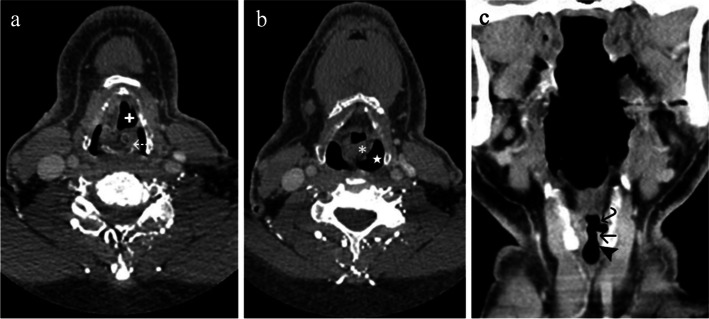
Contrast enhanced CT neck demonstrating indirect features of left RLN palsy. **a** Axial slice demonstrating ipsilateral laryngeal ventricle dilatation ( +) and medial rotation of the arytenoid cartilage ( ←). **b** Axial slice showing medialization of the aryepiglottic fold (*) and mild dilatation of the pyriform sinus (★). **c** Coronal reformat showing reduced bulk and pointed morphology of the vocal cord with loss of normal shouldering in the subglottic arch

A contrast-enhanced CT (CECT) of the neck, chest, abdomen and pelvis was performed in the outpatient setting. The bronchial artery pseudoaneurysm was diagnosed on non-angiographic CT. In addition, the CT neck demonstrated indirect features of left RLN palsy including ipsilateral laryngeal ventricle dilatation, medialization of the aryepiglottic fold, medial rotation of the vocal process of the arytenoid cartilage and mild dilatation of the pyriform sinus (Fig. 1). The coronal reconstruction also showed reduced bulk and pointed morphology of the vocal cord with loss of normal shouldering in the subglottic arch (Fig. 1). The radiological signs were suggestive of RLN palsy in the context of dysphonia and the pseudoaneurysm in the expected location of the left RLN, posteroinferior to the aortic arch involving the tracheo-eosophageal groove. The findings resulted in immediate referral to our tertiary centre for further investigation and management.

**Fig. 2:**
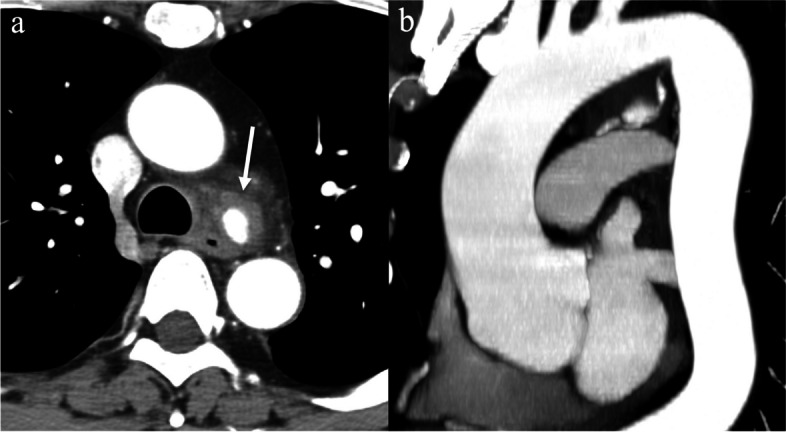
Pre-treatment imaging of the bronchial artery pseudoaneurysm. **a** CT angiogram axial slice demonstrating filling of the pseudoaneurysm between the descending thoracic aorta and trachea with surrounding haematoma. **b** CT angiogram oblique coronal maximal intensity projection (MIP) reconstruction showing the pseudoaneurysm inferior to the aortic arch

Dedicated work-up CT angiogram confirmed a 20 × 22 × 20 mm pseudoaneurysm just inferior to the aortic arch, located 16 mm anteromedial to the proximal descending thoracic aorta, abutting the thoracic oesophagus and trachea (Fig. 2). Two right bronchial arteries were visualised arising orthotopically from the aorta at the T5 level, one coming off an intercostobronchial trunk (ICBT) and the other directly from the aorta, and a single left bronchial artery directly off the aorta (type IV) [3]. The pseudoaneurysm was associated with the right bronchial artery off the ICBT (Fig. 2). There was irregularity and mild dilatation of the vessel just proximal and distal to the pseudoaneurysm and the artery was patent distally extending down to the right main bronchus. It was thought to be a pseudoaneurysm due to irregularity of the parent vessel, surrounding haematoma and lack of atherosclerosis. Nasendoscopy in the emergency department confirmed fixed medialisation of the left vocal cord and no other cause for dysphonia.

**Fig. 3:**
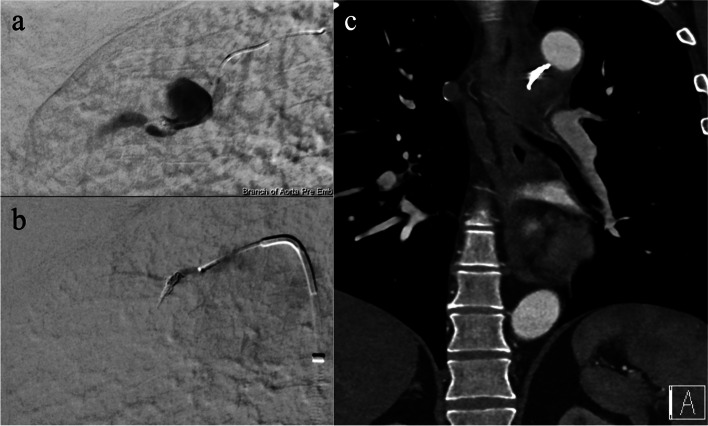
Bronchial artery pseudoaneurysm pre and post embolization. **a** Selective angiogram via a microcatheter in the right bronchial artery showing the pseudoaneurysm. **b** Selective angiogram post coil embolization demonstrating no residual filling in the pseudoaneurysm. **c** Follow-up CT angiogram one day post procedure demonstrating successful exclusion of pseudoaneurysm

After multidisciplinary review, a decision was made for BAE. Right femoral access was obtained under ultrasound guidance and local anaesthesia before placement of a long 6 Fr sheath. The ICBT was selected with a 5 Fr C2 catheter (Cordis, Cardinal Health, Miami Lakes, USA). A 2.4 Fr Progreat microcatheter (Terumo, Tokyo, Japan) was then advanced to the pseudoaneurysm and selective coil embolisation performed. One 2 mm × 2 cm coil and three 2 mm × 3 cm detachable extra soft Smart Coils (Penumbra, Almeda, USA) were placed in the feeding bronchial artery across the pseudoaneurysm neck, successfully occluding the arterial inflow to the pseudoaneurysm (Fig. 3). Tenuous access, vessel irregularity and vasospasm precluded accessing distal to the neck. Completion angiogram and follow-up CT angiogram one day post procedure confirmed successful exclusion with no residual enhancement/flow in the BAP (Fig. 3).

Further investigation post embolisation was unremarkable. His bloods remained normal. Repeated blood cultures were negative and the screen for atypical infections, including tuberculous and non-tuberculous mycobacteria, syphilis, HIV and COVID was negative. The vasculitis screen was negative. Positron emission tomography (PET) showed no significant FDG-avidity to suggest malignancy or infection in the mediastinum or elsewhere. Transthoracic echocardiogram was negative. It was therefore concluded that the BAP was most likely idiopathic, although underlying connective tissue disorder remained a possibility. There was complete resolution of his symptoms by the time of discharge 7 days post embolization and no recurrence of symptoms on outpatient follow-up at 3 months. No follow-up imaging was performed.

## Discussion

The pathogenesis and aetiology of BAPs and BAAs is not well understood [4]. They can be classified according to location, divided into mediastinal, intrapulmonary or both [2]. They are commonly associated with lung disease such as bronchiectasis (including cystic fibrosis), recurrent infection, silicosis, inflammatory lung diseases and lung cancer [5]. Vascular pathologies such as atherosclerosis, hypertension, vasculitis and vascular syndromes such as hereditary haemorrhagic telangiectasia (Osler-Weber-Rendu) are also known predisposing factors [5, 6]. Trauma, iatrogenesis and anticoagulation are other important considerations. However, the most comprehensive review to date by Noberto et al. in 2018 suggested that almost one half of BAP cases (49.1%) are idiopathic [7].

Contained pseudoaneurysms without overt rupture tend to be indolent, presenting either as asymptomatic incidental findings diagnosed on CT or with symptoms such as dysphagia, chest discomfort, cough or vague symptoms that raise suspicion of occult malignancy [8, 9]. Ruptured pseudoaneurysms most frequently present with thoracodynia or haemoptysis [1, 10]. Importantly, BAP rupture can result in life-threatening haemorrhage. Massive haemoptysis, defined as greater than 300 mL within a 24-h period, most commonly comes from a bronchial arterial source (> 90%) and carries a mortality exceeding 50% [11, 12]. The variable size of ruptured BAPs in the literature has led to the inference that diameter is not necessarily an incremental risk factor for rupture [13, 14]. The risk of rupture with false aneurysm is higher than a true aneurysm, although making the distinction is not always possible [1]. For these reasons, timely diagnosis, early referral and urgent treatment is recommended whenever possible, regardless of size or symptomatology [15].

Contrast-enhanced CT tends to be the first line imaging modality to diagnose and characterise BAPs and guide subsequent treatment. Digital subtraction angiography (DSA) is the gold standard for diagnostic confirmation with a sensitivity of 100% compared to CT which is 67% [16]. The main differential diagnoses on imaging would be a saccular aneurysm arising from the aorta, ductus arteriosus, aberrant right subclavian artery or another aortic branch.

Up to 40% of patients with unilateral vocal cord palsy are asymptomatic at the time of diagnosis and this may be further confounded by the presence of other symptoms or other cranial nerve involvement with the underlying disease process [17]. Recovery from Ortner’s syndrome (RLN palsy secondary to cardiovascular aetiologies) has not been well studied. In 85.7% of cases, symptoms resolve within 1 week to 3 years but the chance of recovery likely depends on the degree and duration of RLN palsy [18].

The two main treatment options are endovascular repair or open surgery. Whilst they have a comparable success rate (93.1% vs 90.0% respectively), endovascular techniques are recognised as safer, less invasive, less painful, more selective, lower in cost and offer shorter hospital stays and improved quality of life [7, 15]. Embolisation is generally accepted as first line, most commonly with coils alone or in combination with liquid embolics such as gelatin sponge, polyvinyl acetate (PVA), cynanoacrylate (glue) or onyx [7]. Placement of an aortic stent-graft is another endovascular option but limits access back into the bronchial arteries if retreatment is required, can be problematic in the setting of infection and is associated with increased cost, time and risk [19–21]. Surgery may be considered where the anatomical configuration makes definitive embolisation impossible or at centres that lack the required endovascular expertise [22]. Surgical techniques include open or thoracoscopic resection or ligation (with or without vascular reconstruction), lobectomy or pneumonectomy [23].

This case report highlights a rare case of unruptured BAP presenting with RLN palsy. As with most cases, the presentation was indolent and no underlying cause was found. The pseudoaneurysm was treated early and successfully with coil embolization resulting in complete resolution of the symptoms related to RLN compression.

## Conclusion

BAPs are rare and potentially life-threatening. Whilst the age and idiopathic nature of the case we present is most common for this disease, the presentation with clinical and radiological signs of RLN palsy is atypical and unique. Timely diagnosis and referral to facilitate urgent BAE is essential to prevent potentially serious clinical sequelae. Endovascular treatment in the form of BAE is considered first line.

## Data Availability

Data sharing is not applicable to this article as no datasets were generated or analysed during the current study. Further information can be made available by the authors on request.

## Abbreviations

BAP: Bronchial artery pseudoaneurysm
BAA: Bronchial artery aneurysm
BAE: Bronchial artery embolization
ICBT: Intercostobronchial trunk
RLN: Recurrent laryngeal nerve
ANA: Antinuclear antibodies
ENA: Extractable nuclear antigens
CECT: Contrast-enhanced computed tomography
PET: Positron emission tomography
MRI: Magnetic resonance imaging
